# The relationship between social support and smartphone addiction among Chinese college students: the mediating role of loneliness and the moderating role of meaning in life

**DOI:** 10.3389/fpubh.2025.1671800

**Published:** 2025-09-18

**Authors:** Jialei Liu, Yongli Zhang, Yu Zhang, Qinglei Wang

**Affiliations:** ^1^College of Teachers, Chengdu University, Chengdu, China; ^2^School of Educational Sciences, Northwest Normal University, Lanzhou, China; ^3^School of Management, Northwest Normal University, Lanzhou, China

**Keywords:** social support, smartphone addiction, loneliness, meaning in life, college students

## Abstract

**Background:**

Smartphone addiction has become a significant public health concern among Chinese college students, adversely affecting their academic performance, social interactions, and psychological well-being. Based on the social support buffering hypothesis and compensatory internet use theory, loneliness may mediate the relationship between social support and smartphone addiction, with meaning in life potentially moderating this process.

**Methods:**

A cross-sectional study design was employed. Valid data were collected from 2076 Chinese college students using the Social Support Rating Scale for College Students, the 8-item UCLA Loneliness Scale (ULS-8), the Meaning in Life Questionnaire, and the Smartphone Addiction Scale-Short Version. Data analysis was conducted using SPSS 27 and the PROCESS macro to examine the mediating effect and the moderated mediation effect.

**Results:**

Social support was significantly negatively correlated with smartphone addiction. Loneliness mediated this relationship, accounting for 64.71% of the total effect. Meaning in life significantly moderated the path from loneliness to smartphone addiction. Simple slope analysis indicated that the positive predictive effect of loneliness on smartphone addiction was stronger for individuals with high levels of meaning in life.

**Conclusion:**

Social support may reduce the risk of addiction by alleviating loneliness; however, higher levels of meaning in life may amplify, rather than weaken, the negative effect of loneliness. This study reveals the complex interplay between social and existential factors in addiction and suggests that future interventions should focus on enhancing social support.

## 1 Introduction

In recent years, the Internet penetration rate in China has been steadily increasing. According to statistics, the number of mobile Internet users in China has reached 1.105 billion, and the proportion of Internet users accessing the Internet via mobile phones has reached 99.7% (1). The convenience of smartphones has significantly lowered the barrier to accessing information and enriched people’s life experiences. However, improper use of smartphones has also contributed to the growing issue of smartphone addiction. Smartphone addiction, also referred to as mobile phone dependence or problematic mobile phone use, describes individuals who are excessively engaged in activities mediated by mobile phones, exhibiting a strong and persistent desire for and dependence on mobile phone usage, often accompanied by notable social and psychological impairment (2). Smartphone addiction can negatively impact college students physiologically, psychologically, and socially. It can cause sleep procrastination (3), impair sleep quality (4), and lead students with higher addiction tendencies to experience more negative emotions such as sadness and disgust (5). Smartphone addiction can also adversely affect interpersonal relationships, leading to social barriers (6) and subsequently contributing to social anxiety (7). Research from other countries also supports these findings (8, 9). Unfortunately, smartphone addiction is a common problem. A meta-analysis study found that the overall prevalence of smartphone addiction worldwide is 28.3% (10). The smartphone addiction situation of Chinese college students is also serious (11). Especially during the COVID-19 pandemic, due to home isolation, the smartphone addiction rate is generally high (12), even reaching 63.58% in a study (13). What is more worrying is that the degree of smartphone addiction among Chinese college students has increased year by year (14). The formation of smartphone addiction is affected by multiple factors, including gender, anxiety, depression, loneliness, stress, happiness, social support and psychological resilience (15–18). College is a critical period for individual growth, and high-quality college life is crucial to their future development. Smartphone addiction seriously interferes with college students’ daily life and academic performance (19). Therefore, in-depth exploration of its influencing factors has important theoretical and practical significance for preventing college students’ smartphone addiction and promoting their physical and mental health development.

### 1.1 Social support and smartphone addiction

According to the buffering hypothesis, social support can alleviate the negative effects of adverse stimuli on an individual’s mental health (20). In the absence of adequate social support, the protective buffering effect diminishes, which may result in negative psychological outcomes. Smartphones, equipped with integrated social applications, offer accessible online platforms that can satisfy individuals’ social requirements. Consequently, when an individual perceives a low level of genuine social support, they may feel a heightened motivation to use smartphones to seek alternative social fulfillment or emotional comfort to buffer the negative effects of poor psychological states, which can easily lead to smartphone addiction over time.

Studies have demonstrated a significant negative correlation between social support and smartphone addiction (16). Specifically, when individuals perceive a lack of support from significant others, their risk of becoming overly dependent on mobile phones increases markedly (21). A one-year follow-up survey indicated that early social support can effectively predict later smartphone addiction (22). Intervention studies have also confirmed that enhancing social support can mitigate the severity of smartphone addiction among adolescents (23).

### 1.2 Loneliness and smartphone addiction

Loneliness is defined as a distressing subjective experience that arises from an individual’s perception that their social needs are not fulfilled by the quantity and, particularly, the quality of their social relationships (24). It is a subjectively perceived phenomenon, rather than an objective measure of social isolation. In other words, individuals may appear to have a vibrant social life yet still experience feelings of loneliness; conversely, some may lead a relatively solitary existence without feeling lonely.

The compensatory internet use theory posits that individuals possess an intrinsic motivation to utilize the Internet as a means of alleviating negative emotions in adverse life situations, such as profound loneliness (25). For instance, when a person experiences a lack of social stimulation in their offline life, they may turn to online social interactions to mitigate these negative feelings. Consequently, if the absence of social stimulation persists over an extended period, individuals may increasingly depend on online social interactions for emotional compensation, potentially leading to addiction-like behavior patterns.

Loneliness is a prevalent psychological issue among adolescents globally. A survey indicated that approximately 50% of Americans identify as experiencing loneliness, with Generation Z individuals (born from the mid-1990s to the early 2000s, approximately of college age at the time of the survey) exhibiting higher levels of loneliness according to the UCLA Loneliness Scale compared to other adult age cohorts (26). Research conducted in various countries, including multinational investigations, has similarly documented elevated loneliness levels among college student populations (27, 28). In China, longitudinal data reveal a rising trend in loneliness among college students from 1997 to 2018 (29). Contributing factors to loneliness in this demographic encompass changes in living environments, challenges in adapting to interpersonal relationships, and heightened academic pressures (30), with social support identified as a critical predictor influencing the experience of loneliness (31). Research has demonstrated a significant positive correlation between loneliness and smartphone addiction (32), with loneliness serving as a positive predictor of smartphone addiction among college students (33).

### 1.3 The moderating role of meaning in life

Viktor Frankl posited that the fundamental human drive is the pursuit of meaning in life, a concept he identified as the “will to meaning” (34). The construct of meaning in life pertains to an individual’s understanding and perception of the significance of their existence and the nature of life itself (35). Broadly, meaning in life encompasses both the experience of meaning and the active search for it. Some scholars have advanced a tripartite model that highlights three essential components: coherence, purpose, and significance (36). Empirical research has demonstrated a positive association between meaning in life and the extent to which individuals perceive their interpersonal needs as fulfilled, including their sense of belonging and the experience of closeness and support from family members (37). Conversely, chronic loneliness has been shown to diminish the sense of meaning in life (38), whereas fostering a stronger sense of belonging can enhance it (39).

Existentialism posits that addictive behavior initially serves as a mechanism for individuals to cope with existential anxiety, which encompasses the search for meaning in life, the inevitability of death, concerns related to personal freedom, existential loneliness, and the responsibility for one’s life choices (40). According to logotherapy, a weak sense of purpose and meaning in life can lead to increased boredom (41), making individuals more likely to fill this void with addictive activities, thereby fostering addictive tendencies and behaviors. Numerous studies have demonstrated that a meaning in life is negatively correlated with various addictive behaviors (e.g., smoking addiction (42), drug abuse (43), alcohol dependence (44), and Internet addiction (45)). Additionally, research involving college students has found that meaning in life can significantly and negatively predict smartphone addiction (46–48).

### 1.4 The current study

**Figure 1:**
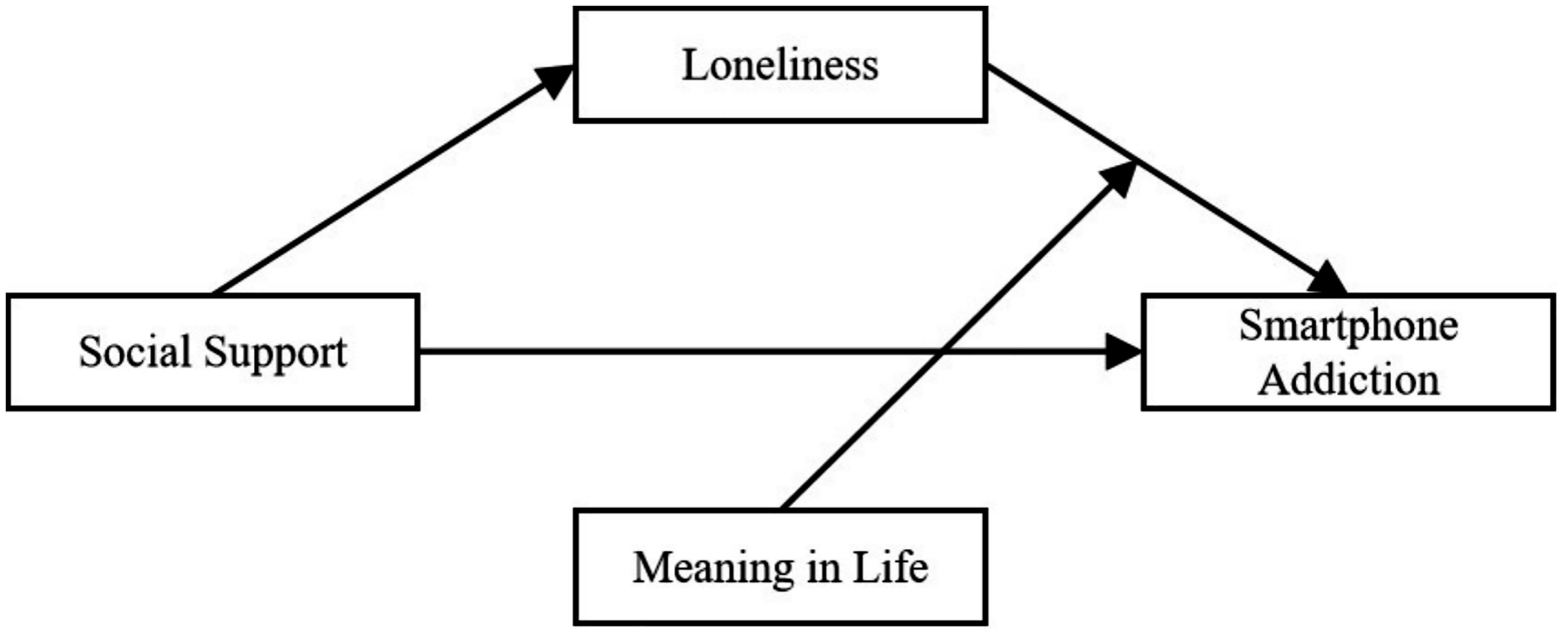
Mediation model. Social support, independent variable; Loneliness, mediator; Meaning in life, moderator; Smartphone addiction, dependent variable. The moderation effect is tested on the path from loneliness to smartphone addiction.

Based on the literature review and research hypotheses, this study further investigates the relationships between social support, loneliness, meaning in life, and smartphone addiction. First, it examines whether loneliness mediates the relationship between social support and smartphone addiction. Secondly, it explores whether the meaning of life moderates the direct and indirect relationship between loneliness and smartphone addiction, with social support as the independent variable and loneliness as the mediating variable. The proposed model is shown in Figure 1, which helps understand the moderating mechanism of meaning in life among social support, loneliness, and smartphone addiction. Based on the literature review, we propose the following hypotheses:

*H1*: Loneliness mediates the relationship between social support and smartphone addiction.

*H2*: Meaning in life moderates the relationship between loneliness and smartphone addiction.

## 2 Methods

### 2.1 Sample and procedure

Participants were recruited through two channels. First, electronic questionnaires were distributed through student counselors at two universities in Sichuan Province, inviting students to volunteer. 1,083 questionnaires were collected via this channel. Second, recruitment information for volunteer participants was posted on RedNote (a well-known Chinese social app) using the first author’s personal account, inviting undergraduate student volunteers to complete the questionnaire. Volunteers were promised compensation upon successful review and submission of the questionnaire. 1,113 questionnaires were collected via this channel. The Tencent Questionnaire platform was used to facilitate preliminary statistics and compensation distribution. Before filling out the questionnaire, participants were informed that the survey was anonymous, would not collect personal information, and that the data would be used solely for academic research and would not be disclosed. Participants provided informed consent before proceeding; those who did not agree were not required to complete the survey. Recruitment took place from April 2 to April 11, 2025. A total of 2,196 questionnaires were collected. As an electronic questionnaire system was used, submissions were not allowed if any items were left blank; thus, the collected data had no missing values. After screening (excluding invalid questionnaires due to patterned responses, excessively short completion times, all answers identical, or incorrect responses to lie detection questions), 2076 valid questionnaires remained, resulting in an effective response rate of 94.54%. To ensure participants did not provide false information due to privacy concerns, we collected information on the type of college attended but did not collect specific details such as the exact college name, major, or age that could identify individuals.

### 2.2 Measures

#### 2.2.1 Loneliness scale

The 8-item UCLA Loneliness Scale (ULS-8) developed by Hays et al. (49) was used, with the Chinese version translated by Zhou Liang et al. (50). This scale consists of 8 items, including 2 reverse-scored items. It uses a 4-point frequency scale (1 = “Never,” 2 = “Rarely,” 3 = “Sometimes,” 4 = “Always”). Higher total scores indicate greater loneliness. In this study, the Cronbach’s *α* coefficient for this scale was 0.861.

#### 2.2.2 Smartphone addiction scale

The Short Version Smartphone Addiction Scale, developed by Kwon et al., was utilized in this study (51). The scale includes a Chinese version and consists of a total of 10 items (52). It is unidimensional, with typical questions such as such as planned work due to smartphone use. It uses a 6-point Likert rating, where 1 represents strongly disagree and 6 represents strongly agree. The Cronbach’s *α* coefficient for this scale in the current study was 0.941.

#### 2.2.3 Social support rating scale for college students

The College Student Social Support Rating Scale developed by Ye Yuemei and Dai Xiaoyang was used (53). Based on the three-factor model of social support (subjective support, objective support, and support utilization), this scale contains 17 items measuring subjective support (e.g., “When facing a dilemma, I actively seek help from others”), objective support (e.g., “I can rely on family or relatives/friends when encountering difficulties”), and support utilization (e.g., “I often receive care and support from classmates and friends”). It uses a 5-point rating method (1 = “Does not conform,” 5 = “Conforms”). In this study, the Cronbach’s *α* coefficient for this scale was 0.963.

#### 2.2.4 Meaning of life questionnaire

The Meaning in Life Questionnaire (MLQ) developed by Steger et al. was used (35), with the Chinese version (C-MLQ) revised by Wang Mengcheng et al. (54). Based on Frankl’s theory of meaning and the definition of meaning in life, Steger described meaning in life through two dimensions: presence of meaning (e.g., “I understand my life’s meaning”) and search for meaning (e.g., “I am looking for something that makes my life feel meaningful”). Thus, this scale includes these two dimensions with a total of 10 items. It uses a 7-point Likert scale (from 1 = “Very untrue” to 7 = “Very true”), with item 9 being reverse-scored. In this study, the Cronbach’s *α* coefficient for this scale was 0.932.

### 2.3 Statistical analysis

Data analysis was performed utilizing SPSS version 27 and the PROCESS macro. Initially, Harman’s single-factor test was applied to assess the presence of common method bias. Subsequently, the normality of the distribution for each variable was evaluated. Considering the distribution characteristics of the data, select suitable statistical techniques to examine differences in demographic variables as well as in smartphone addiction, sense of life meaning, loneliness, and social support. Additionally, identify and apply the appropriate correlation coefficient to assess the bivariate relationships among all research variables. Finally, the hypothesized model was tested using Hayes’s PROCESS macro version 4.2, specifically employing Model 4 and Model 14. Bootstrap sampling with 5,000 iterations was conducted to estimate 95% confidence intervals (CIs) for mediation and moderation effects. An effect was deemed statistically significant if the 95% confidence interval, defined by the lower level confidence interval (LLCI) and upper level confidence interval (ULCI), did not include zero.

## 3 Results

### 3.1 Common method bias test

This study employed the Harman single-factor test method. The results indicated that the KMO value was 0.978, the significance of the Bartlett test was *p* < 0.000, and the initial eigenvalues of the five factors were greater than 1. The explained variance of the first common factor was 39.773%, which is below the critical threshold of 40% (55). Therefore, it can be concluded that there was no significant common method bias in this study.

### 3.2 Preliminary analysis

All participants were current college students, comprising 1,147 males (55.25%) and 929 females (44.75%). Tests for multicollinearity, with smartphone addiction as the dependent variable, showed that the variance inflation factor (VIF) values for social support, loneliness, and meaning in life were all below 3, indicating no serious multicollinearity. Kolmogorov–Smirnov tests, as well as skewness and kurtosis tests, indicated that the scales were not normally distributed. Therefore, the Mann–Whitney U test was used for dichotomous variables (gender, only child status) to examine significant differences on the scales (Z-values are reported in Table 1), and the Kruskal-Wallis test was used for multi-categorical variables. Descriptive statistics for demographic variables and results of difference tests concerning smartphone addiction, meaning in life, loneliness, and social support are presented in Table 1. The findings demonstrated significant variations in smartphone addiction, meaning in life, loneliness, and social support across different family income levels. Additionally, college type was associated with significant differences in smartphone addiction, loneliness, and social support. Family structure was linked to significant differences in loneliness, social support, and meaning in life. Academic year was related to differences in smartphone addiction and loneliness. Gender differences were observed solely in loneliness, whereas only child status was significantly associated only with smartphone addiction.

**Table 1:** Demographic variables.

Variables	*n*	%	Smartphone addiction	Meaning in life	Loneliness	Social support
Gender						
Female	929	44.75				
Male	1,147	55.25				
Z value			−0.339	1.943	−2.912**	−0.112
College type						
985, 211, University of Chinese Academy of Sciences	733	35.31				
Other “Double First-Class” universities	560	26.97				
Other undergraduate	783	37.72				
*H* value			7.418*	3.341	14.981***	50.064***
Grade						
Freshman	397	19.12				
Sophomore year	677	32.61				
Junior year	691	33.29				
Senior year	311	14.98				
*H* value			11.693**	4.631	14.267**	45.441
Only child or not						
Yes	1,272	61.27				
No	804	38.73				
*Z* value			1.612	96.353***	76.343***	48.268***
Family structure						
Two-parent family	1727	83.19				
Single-parent families	254	12.24				
Reconstituted family	71	3.42				
Grandparents	18	0.87				
Others	6	0.29				
*H* value			2.596**	−0.055	0.185	−0.738
Annual household income (RMB)						
Below 10,000	130	6.26				
10,000-20,000	200	9.63				
20,000-50,000	316	15.22				
50,000-100,000	403	19.41				
100,000–200,000	585	28.18				
200,000–500,000	299	14.40				
More than 500,000	143	6.89				
*H* value			36.026***	156.154***	247.153***	242.671***

***p < 0.001; **p < 0.01; *p < 0.05. *n*, sample size; *Z* value, *Z* test value converted from Mann–Whitney *U* test value; *H* value, Kruskal-Wallis test value; “985” refers to 39 universities supported by the Chinese government since 1998 to create world-class universities and high-level disciplines. “211” refers to approximately 112 universities and key disciplines prioritized for development in the 21st century, initiated in 1995. “Double First-Class” refers to universities promoted since 2017 to develop world-class universities and disciplines, comprising 147 institutions. The “Double First-Class” list includes all “211” universities, and the “211” list includes all “985” universities.

Given that the data did not follow a normal distribution, Spearman’s rank correlation analysis was employed to assess the relationships among the observed variables. Table 2 presents the Spearman correlation matrix for all observed variables. Correlation analysis indicated that social support was significantly negatively correlated with smartphone addiction (*r* = −0.365, *p* < 0.01) and loneliness (*r* = −0.766, *p* < 0.01), and significantly positively correlated with meaning in life (*r* = 0.490, *p* < 0.01). Loneliness was significantly positively correlated with smartphone addiction (*r* = 0.434, *p* < 0.01) and significantly negatively correlated with meaning in life (*r* = −0.589, *p* < 0.01). Meaning in life was significantly negatively correlated with smartphone addiction (*r* = −0.365, *p* < 0.01).

**Table 2:** Descriptive statistics and correlations among all observed variables.

Variable	1	2	3	4
1. Smartphone addiction	1			
2. Meaning in life	−0.365^**^	1		
3. Loneliness	0.434^**^	−0.589^**^	1	
4. Social support	−0.365^**^	0.490^**^	−0.766^**^	1

**p < 0.01.

### 3.3 Mediation model test

Based on the results of the differences tests between demographic variables and the four scales, four variables that showed significant differences in smartphone addiction—college type, grade, only child status, and annual household income—were included as control variables. Using Model 4 in the PROCESS macro, the mediating role of loneliness in the relationship between social support and smartphone addiction was tested. All study variables were mean-centered before analysis. The results are shown in Tables 3, 4. Table 3 shows that social support significantly negatively predicted smartphone addiction (*β* = −0.323, t = −20.889, *p* < 0.001) and loneliness (*β* = −0.243, *t* = −51.020, *p* < 0.001). Loneliness significantly positively predicted smartphone addiction (*β* = 0.859, *t* = 12.496, *p* < 0.001). The R^2^ values range from 0 to 1, with values closer to 1 indicating better model fit. According to Table 3, the R^2^ for the mediation model (including the mediator) was 0.239 (*F* = 108.424, *p* < 0.001), and the R^2^ for the direct effect model (without the mediator) was 0.182 (*F* = 91.987, *p* < 0.001). The higher R^2^ for the mediation model suggests the presence of a mediation effect. According to Table 4, the 95% confidence interval for the indirect effect was [−0.243, −0.177], not including zero, indicating a significant mediation effect. The total effect of social support was −0.323, and the indirect effect through loneliness was −0.209. As the 95% confidence intervals for both did not include zero, loneliness mediates the relationship between social support and smartphone addiction, accounting for 64.71% of the total effect. Hypothesis 1 was supported.

**Table 3:** Mediation model.

Outcome variable	Predictor variables	*R* ^2^	*F*	*β*	SE	*t*
Smartphone addiction	Constant	0.182	91.987***	48.030	1.588	30.249***
	Social support			−0.323	0.015	−20.889***
	School type			−0.333	0.313	−1.065
	Grade			0.754	0.269	2.801**
	Only child or not			1.231	0.541	2.273*
	Annual household income			1.067	0.168	6.331***
Loneliness	Constant	0.593	603.066***	34.755	0.489	71.011***
	Social support			−0.243	0.005	−51.020***
	School type			−0.286	0.096	−2.967**
	Grade			−0.165	0.083	−1.985*
	Only child or not			−0.113	0.167	−0.677
	Annual household income			−0.336	0.052	−6.470***
Smartphone addiction	Constant	0.239	108.424***	18.162	2.839	6.398***
	Social support			−0.114	0.022	−5.088***
	Loneliness			0.859	0.069	12.496***
	School type			−0.087	0.302	−0.289
	Grade			0.896	0.260	3.446***
	Only child or not			1.328	0.522	2.542*
	Annual household income			1.355	0.164	8.258***

***p < 0.001; **p < 0.01; *p < 0.05. *R*^2^, coefficient of determination; *F*, *F* statistic; *β*, standardized regression coefficient; SE, standard error; *t*, *t* statistic.

**Table 4:** Results of the mediation effect test.

	Effect value	SE	LLCI	ULCI	Effect value proportion
Total	−0.323	0.015	−0.354	−0.293	
Direct	−0.114	0.022	−0.158	−0.070	35.29%
Indirect	−0.209	0.017	−0.243	−0.177	64.71%

LLCI, lower limit of confidence interval; ULCI, upper limit of confidence interval.

### 3.4 The moderating effect of meaning in life

**Figure 2:**
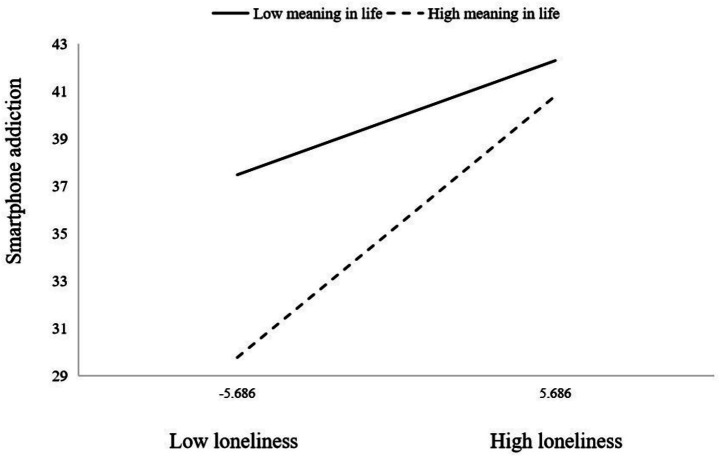
The moderating effect of meaning in life on loneliness and smartphone addiction.

Controlling for college type, academic grade, only-child status, and annual household income, Model 14 of the PROCESS macro was employed to examine smartphone addiction as the dependent variable, social support as the independent variable, loneliness as the mediator, and meaning in life as the moderator. The findings, presented in Table 5, revealed that the interaction between meaning in life and loneliness significantly predicted smartphone addiction (*β* = 0.021, t = 6.267, *p* < 0.001), indicating that meaning in life moderates the relationship between loneliness and smartphone addiction. To further elucidate this moderation effect, the association between loneliness and smartphone addiction was plotted at low (M-1SD) and high (M + 1SD) levels of meaning in life (see Figure 2). Simple slope analyses demonstrated that loneliness significantly predicted smartphone addiction at both high (*β* = 0.971, *p* < 0.001) and low (*β* = 0.424, *p* < 0.001) levels of meaning in life; however, the effect was more pronounced at higher levels of meaning in life. These results suggest that meaning in life moderates the influence of loneliness on smartphone addiction, thereby supporting Hypothesis 2.

**Table 5:** Moderation model.

Outcome variable	Predictor variables	R^2^	*F*	*β*	SE	*t*
Loneliness	Constant	0.593	603.066***	16.678	0.489	34.076***
	Social support			−0.243	0.005	−51.020***
	School type			−0.286	0.096	−2.967**
	Grade			−0.165	0.083	−1.985*
	Only child or not			−0.113	0.167	−0.677
	Annual household income			−0.336	0.052	−6.470***
Smartphone addiction	Constant	0.268	94.816***	32.680	32.71	17.361
	Social support			−0.093	−0.095	−4.273***
	Loneliness			0.689	0.697	9.654***
	Meaning in life			−0.183	−0.179	−7.522***
	Loneliness × Meaning in life			0.021	0.021	6.183***
	School type			−0.049	−0.032	−0.109
	Grade			0.946	0.937	3.673***
	Only child or not			1.240	1.253	2.446*
	Annual household income			1.510	1.507	9.288***

***p < 0.001; **p < 0.01; *p < 0.05.

## 4 Discussion

This study constructed a moderated mediation model involving social support, smartphone addiction, loneliness, and meaning in life to explore the internal mechanism through which social support influences smartphone addiction. The results indicated that loneliness mediates the relationship between social support and smartphone addiction. Meaning in life significantly moderated the direct effect of loneliness on smartphone addiction, with the moderating effect manifesting such that high meaning in life strengthened (rather than weakened, as might be intuitively expected) the positive predictive effect of loneliness on smartphone addiction.

### 4.1 Relationship between social support and smartphone addiction

This study found that social support is a significant predictor of smartphone addiction, which aligns with the conclusions of previous research (18). This finding strongly supports the buffering hypothesis. According to this hypothesis, students with higher levels of social support can mitigate the negative impact of stressful events or environments through resources provided by their social networks, such as family and friends. In contrast, students with lower social support, due to the lack of sufficient external support resources, are unable to rely on others to alleviate the stress caused by adverse events or environments. When confronted with such challenges, they are more likely to seek alternative coping mechanisms to escape negative emotions or situations. Smartphones, being highly accessible and portable, offer a wealth of information and virtual social interactions, making them a common tool for these escape behaviors. Consequently, individuals who perceive low social support are more likely to excessively rely on smartphones to compensate for the absence of real support or to relieve stress, thereby increasing their risk of smartphone addiction.

### 4.2 The mediating role of loneliness

The results indicated that loneliness mediated the relationship between social support and smartphone addiction, supporting the compensatory Internet use theory. According to this theory, internet addiction can serve as a coping mechanism for individuals facing adverse living conditions or seeking to alleviate negative emotional states (56). Those who perceive low levels of social support often struggle to establish interpersonal relationships that fulfill their needs in real life, making them more susceptible to feelings of intense loneliness. Various social applications available on smartphones facilitate more convenient virtual social interactions. Due to their accessibility and controllability, these virtual interactions may be viewed as a compensatory measure for the lack of genuine social engagement and, in some cases, may even be perceived as a higher-quality or safer alternative. Consequently, individuals are more likely to mitigate feelings of loneliness and satisfy their interpersonal needs by immersing themselves in virtual social interactions. However, a long-term reliance on these virtual interactions, rather than real life connections, may not only impede the development of essential social skills but could also lead to excessive dependence on and desire for mobile phone social functions, ultimately resulting in smartphone addiction. It is important to note that this escapist use of smartphones may create a vicious cycle: excessive engagement in virtual social interactions can exacerbate social alienation and loneliness in real life.

### 4.3 The moderating role of meaning in life

The results of this study indicate a negative correlation between meaning in life and smartphone addiction, consistent with previous research (48, 57). Simultaneously, meaning in life played a significant moderating role in the relationship between loneliness and smartphone addiction. Specifically, the positive predictive effect of loneliness on smartphone addiction varied across different levels of meaning in life. When individuals had lower levels of meaning in life, the effect of loneliness on smartphone addiction, although significant, was relatively weaker. Conversely, when individuals had higher levels of meaning in life, the effect of loneliness on smartphone addiction was stronger. This finding may suggest an “amplification” mechanism rather than a buffering one. Previous research indicates that the relationship between feelings of loneliness and a sense of life’s meaning is rather complex (58). For instance, reflection serves as a moderating factor between loneliness and meaning in life, with individuals exhibiting high levels of reflection demonstrating a weaker association between the two constructs (59). Thus, while loneliness and meaning in life are distinct yet interdependent (60), their interaction may be influenced by additional variables, potentially leading to tension between them. Individuals with a strong sense of meaning in life typically pursue and expect greater depth, purpose, and higher-quality interpersonal relationships. When such individuals experience loneliness due to insufficient social connections, a conflict arises. In such cases, the feeling of loneliness may transcend a mere emotional state and manifest as a distressing existential void that contradicts their perceived meaning in life. To alleviate this profound sense of dissonance, they may be more motivated to seek immediate and convenient alternative satisfaction. The virtual social interaction, abundant information, and entertainment content provided by smartphones become a readily available compensatory channel. Therefore, their compensatory usage motivation is stronger, causing loneliness to be more easily translated into addictive behavior. Conversely, for individuals with lower meaning in life, their investment in and expectations for life might be lower, and their need for high-quality interpersonal connections might be relatively weaker. Thus, even when feeling lonely, their intrinsic motivation to compensate via their phone to fill an existential “meaning vacuum” is relatively insufficient, resulting in a weaker driving effect of loneliness on addictive behavior. Therefore, within this specific model context, meaning in life does not act as a simple protective factor but rather as an “amplifier” that increases sensitivity to the existential threat posed by loneliness, thereby strengthening rather than weakening the path toward compensatory addictive behavior. This provides a new perspective for understanding the complex role of meaning in life in addictive behaviors.

## 5 Limitations and future research

When interpreting the results of this study, several potential limitations should be acknowledged. First, the data were primarily obtained from two universities and social media recruitment groups, which may limit regional representativeness and introduce self-selection bias. Second, this study employed a cross-sectional design, which restricts the ability to establish causal relationships among variables. Future research should adopt multi-time-point longitudinal tracking or experimental or quasi-experimental designs to more clearly delineate the temporal dynamics and causal pathways among social support, loneliness, meaning in life, and smartphone addiction. Third, this study relied mainly on self-report measures for data collection, which may result in recall bias and social desirability effects, particularly concerning sensitive topics such as addictive behavior. Future studies should incorporate objective behavioral data (e.g., screen time, application usage logs) to enable multi-method assessments and enhance research validity. Fourth, this study focused solely on the effects of social support, loneliness, and meaning in life on smartphone addiction, without accounting for other potential factors, which may limit the explanatory power of the model. Finally, other studies have indicated that meaning in life varies with age (61); therefore, the conclusions of this study may not be generalizable to other age groups.

Future research should adopt more rigorous longitudinal or experimental designs, expand sampling sources to improve representativeness and diversity, integrate both subjective and objective measures of key variables, include a broader range of influencing and control variables, and further investigate the complex relationship between social support and smartphone addiction.

## 6 Conclusion

Despite the aforementioned limitations, this study holds significant importance. By employing a moderated mediation model, it delves into the mechanisms linking social support, loneliness, meaning in life, and smartphone addiction. Firstly, social support not only directly negatively predicts smartphone addiction among college students but also exerts an indirect influence through the mediating role of loneliness. This finding validates the social support buffering hypothesis and the compensatory internet use theory, indicating that students lacking real world social support are more likely to experience loneliness and subsequently resort to excessive smartphone use for emotional comfort and social compensation, ultimately leading to addiction. Secondly, a key finding of this study is the complex role of meaning in life. The study found that meaning in life moderates the effect of loneliness on smartphone addiction, but the direction of moderation was contrary to what might be initially expected: high meaning in life strengthened the positive predictive effect of loneliness on smartphone addiction. This suggests that for students pursuing life meaning and purpose, loneliness in reality (implying a lack of meaning) induces stronger psychological discomfort, driving them to use their phones more frequently to cope with this state, thereby exacerbating the risk of addiction. Based on the results, mental health initiatives in universities should focus on building robust social support systems, enhancing students’ sense of belonging through group counseling, club activities, etc., to alleviate loneliness at its source. For student groups with high meaning in life, special attention should be given to guide them toward constructive ways (e.g., participating in volunteer services, deep reading, developing hobbies) rather than passive immersion in smartphones to explore and realize life meaning, helping them translate their sense of meaning into realistic, healthy actions.

## Data Availability

The original contributions presented in the study are included in the article/Supplementary material, further inquiries can be directed to the corresponding author/s.
